# Production of four *Neurospora crassa* lytic polysaccharide monooxygenases in *Pichia pastoris* monitored by a fluorimetric assay

**DOI:** 10.1186/1754-6834-5-79

**Published:** 2012-10-26

**Authors:** Roman Kittl, Daniel Kracher, Daniel Burgstaller, Dietmar Haltrich, Roland Ludwig

**Affiliations:** 1 Department of Food Sciences and Technology, Food Biotechnology Laboratory, BOKU – University of Natural Resources and Life Sciences, Muthgasse 18, Vienna, 1190, Austria

## Abstract

**Background:**

Recent studies demonstrate that enzymes from the glycosyl hydrolase family 61 (GH61) show lytic polysaccharide monooxygenase (PMO) activity. Together with cellobiose dehydrogenase (CDH) an enzymatic system capable of oxidative cellulose cleavage is formed, which increases the efficiency of cellulases and put PMOs at focus of biofuel research. Large amounts of purified PMOs, which are difficult to obtain from the native fungal producers, are needed to study their reaction kinetics, structure and industrial application. In addition, a fast and robust enzymatic assay is necessary to monitor enzyme production and purification.

**Results:**

Four *pmo* genes from *Neurospora crassa* were expressed in *P. pastoris* under control of the AOX1 promoter. High yields were obtained for the glycosylated gene products PMO-01867, PMO-02916 and PMO-08760 (>300 mg L^-1^), whereas the yield of non-glycosylated PMO-03328 was moderate (~45 mg L^-1^). The production and purification of all four enzymes was specifically followed by a newly developed, fast assay based on a side reaction of PMO: the production of H_2_O_2_ in the presence of reductants. While ascorbate is a suitable reductant for homogeneous PMO preparations, fermentation samples require the specific electron donor CDH.

**Conclusions:**

*P. pastoris* is a high performing expression host for *N. crassa* PMOs. The *pmo* genes under control of the native signal sequence are correctly processed and active. The novel CDH-based enzyme assay allows fast determination of PMO activity in fermentation samples and is robust against interfering matrix components.

## Background

Hydrolysis is still a major cost factor in the production of second-generation biofuels from lignocellulosic biomass, considering the costs of currently available enzyme mixtures. Until recently, only hydrolytic enzymes were thought to play a role in the degradation of recalcitrant cellulose and hemicelluloses to fermentable sugars. The finding that enzymes from glycosyl hydrolase family 61 (GH61) in combination with the flavocytochrome cellobiose dehydrogenase (CDH) enhance the action of hydrolytic enzymes added a new dimension to the classical concept of cellulose degradation, as recently reviewed by Horn et al. [1]. These copper-dependent enzymes were shown to cleave cellulose by an oxidative mechanism provided that reduction equivalents from CDH or low molecular weight reducing agents (e.g. ascorbate) are available [2-4]. GH61 enzymes with this confirmed activity have been termed polysaccharide monooxygenases (PMOs) [3] or, to indicate the reaction mechanism more specificly, lytic PMOs [1]. Recently, it was shown that enzymes of the structurally similar CBM33 family are also capable of cleaving cellulose. Unlike lytic PMOs they are mainly found in bacteria, but also in other eukaryotes [5].

The CDH/PMO system was shown to improve the degradation of cellulose in combination with cellulases in several studies [2-4,6,7]. In the proposed reaction mechanism CDH donates an electron via its cytochrome domain to the type-2 copper in the PMO active site. There, oxygen is partially reduced and attacks the pyranose ring of the glucose moieties at the C-1 (class-1 PMOs) or C-4 (class-2 PMOs) position, thereby destabilizing the adjacent glycosidic bond and breaking it by an elimination reaction [3,8]. The occurrence of *gh61 (pmo)* genes has been confirmed in many cellulolytic fungi. In some genomes, *gh61* genes even outnumber cellulase genes [9-11]. It remains to be elucidated whether all of these encoded enzymes have PMO activity, but their large number emphasizes the importance of oxidative cellulose cleavage. In the cellulolytic ascomycete *Neurospora crassa*, 2 *cdh* genes and 14 *pmo* genes are present. When the fungus is grown on *Miscanthus* one *cdh* gene and 8 *pmo* genes are upregulated [11]. In our study the expression of four of these *pmo* genes in *Pichia pastoris* is investigated. Only few examples of heterologous expression of *pmo* genes are hitherto known. A PMO from *Thermoascus aurantiacus* was expressed in *Trichoderma reesei* by Novozymes [6], and three PMOs (from *Phanerochaete chrysosporium, Sporotrichum thermophile* and *Aspergillus kawachii*) were produced in *P. pastoris* with the α-factor signal sequence for secretion [7,12,13]. Although the expressions and purifications were successful, none of these studies reported production or purification yields. One reason for that is certainly the lack of a fast and robust assay for PMO activity. The assays currently used to determine PMO activity are based on a lengthy incubation of the PMO together with CDH or a reductant such as ascorbate and the polymeric cellulosic substrates Avicel, phosphoric acid swollen cellulose (PASC), carboxymethyl cellulose (CMC) or nano-fibrillated cellulose at elevated temperatures (37°C – 50°C) for several hours or up to three days. Subsequently, the reaction products are analyzed by MALDI-TOF/MS, HPAEC or LC/MS [3,5,6,12-14]. While these time-consuming and labor-intensive assays are a good choice for investigating the reaction mechanism and substrate specificity, they are of little use for monitoring fermentation processes or the progress of a purification procedure. The proposed reaction mechanism [3] points to the possibility that hydrogen peroxide is generated as a by-product of a futile side reaction, which might occur in the absence of cellulose as a substrate. Li et al. [15] found that peroxide can be modeled well in an electron density at the coordination site of the type-2 copper in *N. crassa* PMO-3 [GenBank: NCU07898]. In this work, we measure the oxygen reducing side reactivity of PMOs by quantifying the formation of hydrogen peroxide. Based on this observation a fast and robust enzymatic assay for PMO activity was developed and tested on the expression and purification of four PMOs from *N. crassa*.

## Results and discussion

### Polysaccharide monooxygenase activity assay

**Figure 1:**
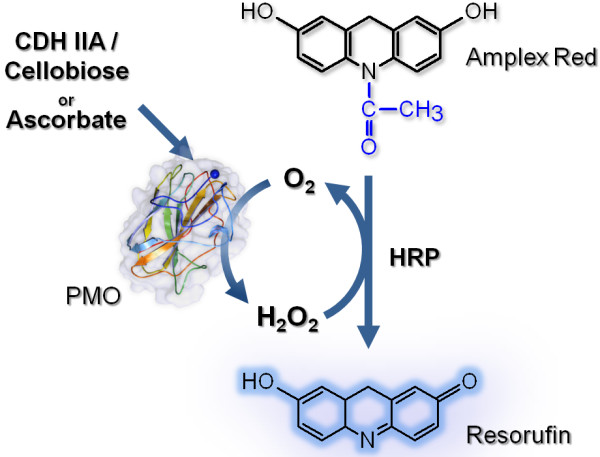
**Schematic representation of the PMO activity assay.** Ascorbate or CDH reduce the type-2 copper in the PMO, which activates molecular oxygen by a one electron reduction. The released H_2_O_2_ is detected by the HRP coupled conversion of Amplex Red to resurofin.

This newly developed assay is based on an easily accessible side-reaction of PMO. The reduction of O_2_ to H_2_O_2_ by PMO has not been reported so far and is not the proposed in vivo function, but is thought to occur as a futile side-reaction, especially in the absence of the natural substrate cellulose. By adding different amounts of PMO to the assay mixture we could prove that the formation of H_2_O_2_ is directly proportional to the PMO concentration. The formation of H_2_O_2_ depends on the availability of a suitable reductant for the PMO type-2 copper center. In this study, ascorbate or the proposed natural interaction partner of PMOs – CDH together with cellobiose – was used as reductant/electron donor. The proposed reaction scheme is given in Figure 1.

**Figure 2:**
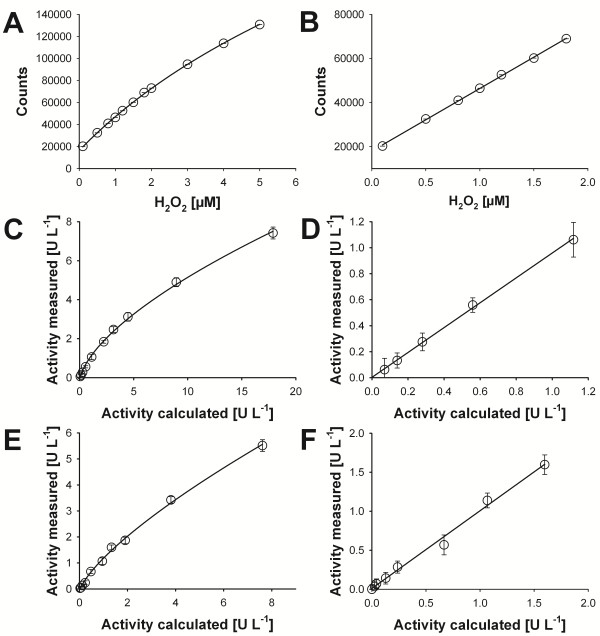
**Calibration and response curves.** (**A**) H_2_O_2_ full concentration range; (**B**) H_2_O_2_ linear concentration range; (**C**) Ascorbate-driven assay: full concentration range fitted by a 2 parameter power function (y = a⋅x^b^); (**D**) Ascorbate-driven assay: linear concentration range (y = a⋅x); (**E**) CDH-driven assay: full concentration range fitted by a 2 parameter power function; (**F**) CDH-driven assay: linear concentration range. Error bars indicate standard deviations calculated from five repetitions.

**Figure 3:**
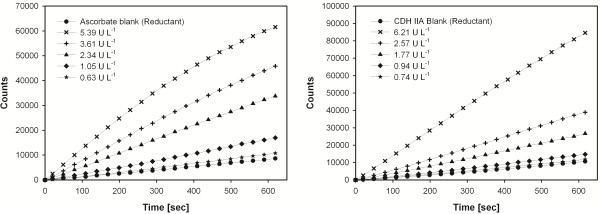
**Background activities of reductants and total activities in the presence of various concentrations of PMO (PMO-02916).** Assays were performed under standard conditions.

H_2_O_2_ formed in this reaction was selectively and sensitively detected with the Amplex Red/horseradish peroxidase reaction [16]. The stability of the resorufin fluorescence was tested for 40 min and shown to be stable much longer than the assay time of 10 min (Additional file 1). The fluorimetric signal response is linear for a range from 0.1 – 2 μM H_2_O_2_ with a slight offset from background fluorescence (y = a⋅x + y_0_). It can be very well approximated by a 3 parameter logarithmic function (y = y_0_+a⋅ln(x-x_0_)) up to a PMO concentration of 5 μM (R^2^ = 0.99) (Figure 2 A, B). The direct proportionality between the concentration of added PMO and measured H_2_O_2_ was observed for the ascorbate- as well as for the CDH-driven assay. A linear range was found for up to 1.2 U L^-1^ of PMO activity when using ascorbate and 1.8 U L^-1^ when using CDH (Figure 2 C-F). The background activity caused by the formation of H_2_O_2_ by autooxidation of ascorbic acid as well as by the weak H_2_O_2_ producing activity of CDH is significant (Table 1). Therefore, activities of blank reactions without PMOs were subtracted from the overall activities measured in the presence of PMOs. If possible, the fluorescence should be continuously measured (no end-point determination) to increase the correctness and precision of the measurement (Figure 3).

**Table 1:** Contribution of background activities of CDH IIA and ascorbic acid, respectively, to the total activity measured with various concentrations of PMO (PMO-02916)

**Assay**	**PMO concentration**	**H**_**2**_**O**_**2**_**production**	**PMO activity**	**Background activity**
	**[mg mL**^**-1**^**]**	**[μmol L**^**-1**^**min**^**-1**^**]**	**[μmol L**^**-1**^**min**^**-1**^**]**	**[%]**
**Ascorbate**	2.87	5.39 ± 0.23	4.90	9.1
	1.43	3.61 ± 0.22	3.12	13
	0.72	2.34 ± 0.05	1.85	21
	0.18	1.05 ± 0.01	0.56	47
	0.02	0.63 ± 0.02	0.14	78
Blank	0	0.49 ± 0.02	0	0
**CDH IIA**	5.74	6.21 ± 0.22	5.51	11
	1.43	2.57 ± 0.11	1.87	27
	0.72	1.77 ± 0.11	1.07	40
	0.18	0.94 ± 0.05	0.24	74
	0.02	0.74 ± 0.05	0.04	94
Blank	0	0.70 ± 0.04	0	0

We employed CDH IIA from *N. crassa* for this assay, which is the major CDH in the *N. crassa* secretome [17] and which was recombinantly produced in *P. pastoris*[18]. A possible direct interaction of PMO with cellobiose, the electron donor of CDH, was tested by replacing cellobiose by lactose. Lactose interacts almost equally well with *Nc*CDH IIA [18] and did not alter the peroxide formation by PMOs.

PMO activity in fermentation samples can only be determined with the CDH-driven assay, since the strong reducing agent ascorbate interacts unspecifically with other oxidases present in the *P. pastoris* culture supernatant (e.g. alcohol oxidase 1) and media components, generating a strong background signal. To exclude the effects of media components (e.g. copper ions) on the CDH-based assay, sample aliquots were additionally centrifuged in mini-spin columns with 10-kDa cut-off, and the blank activity of the permeate was subtracted. A possible interference by proteins in the *P. pastoris* culture supernatant on the assay was examined by measuring background activities of culture supernatant and its permeate (10 kDa cut-off) of two similar fermentations of a *P. pastoris* strain without PMO. The media components in the permeate increase the blank activity significantly (1.6 times). The increase of the background H_2_O_2_ production by the culture supernatant is 1.7 fold (Additional file 2). This shows that compounds with a molecular mass above 10 kDa (proteins) have also a limited effect. The estimated error for using the permeate as blank experiment instead of the full sample matrix is 8-10%, which seems acceptable for the monitoring of PMO activity in fermentations.

### Recombinant production of PMO-01867, PMO-02916, PMO-03328 and PMO-08760

**Figure 4:**
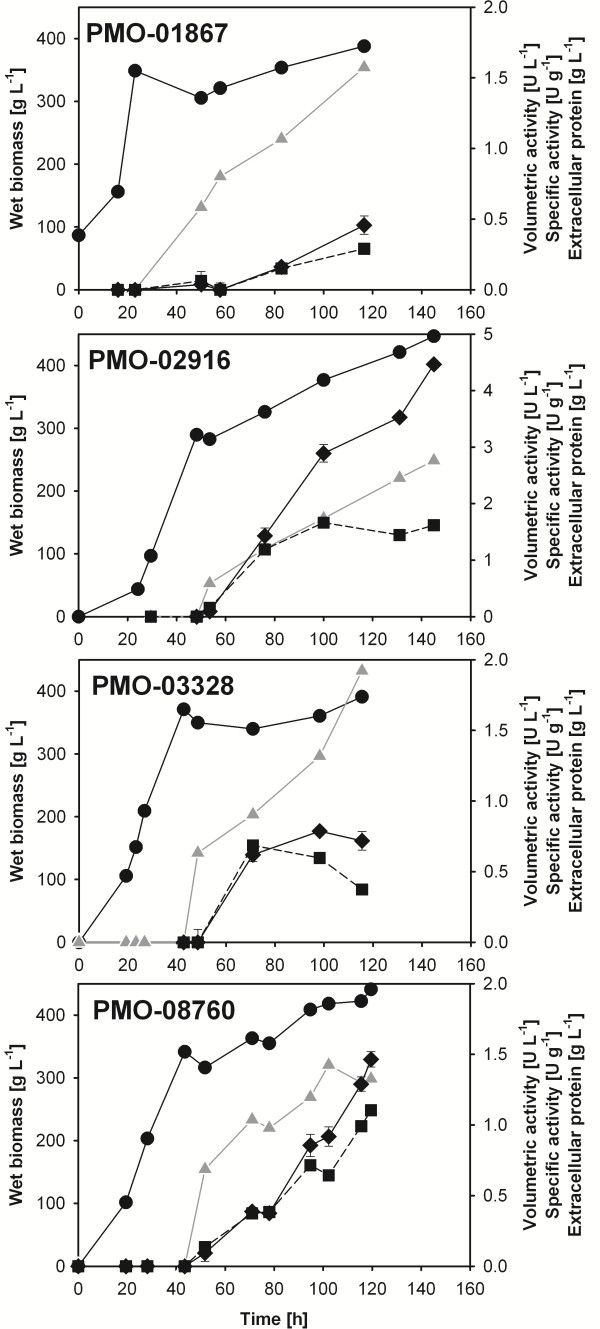
**Production of PMOs in*P. pastoris.*** Circles, wet biomass; triangles, extracellular protein concentration; diamonds, activity; squares, specific activity.

**Figure 5:**
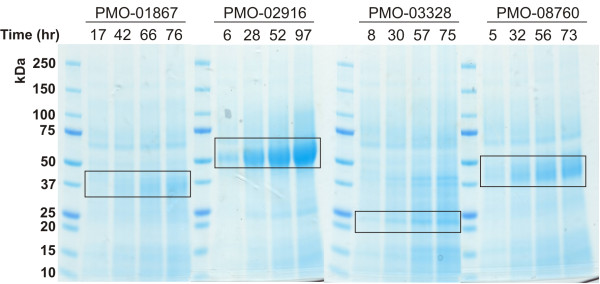
**SDS-PAGE of PMO fermentation samples after induction (methanol induction = 0 h).** The formation of PMO is visible by increasing band strength (boxes).

Four *pmo* genes from *N. crassa* were selected for this study based on the phylogenetic analysis of Li et al. [15] and Sygmund et al. [18]. The closely related genes NCU08760 and NCU01867 were chosen, since both include a family 1 cellulose-binding module (CBM) and are classified as type-1 PMOs [15]. A transcriptome analysis of *N. crassa* showed that the transcription level of NCU08760 increased 107-fold when grown on *Miscanthus* compared to minimal medium*,* whereas the transcription level of NCU01867 remained almost unchanged [11]. Additionally, native NCU08760 was purified from *N. crassa* previously and its function as polysaccharide monooxygenase was confirmed [3,8]. Another type-1 PMO [GenBank:NCU03328] was chosen as an example for a PMO without CBM that is also induced, but to a minor extent (26-fold). NCU02916 is classified as a type-2 PMO, has a CBM and is induced during growth on *Miscanthus* (85-fold). The cDNAs of these four GH61 enzymes were commercially synthesized including their native signal sequences and were codon-optimized for expression in *P. pastoris*. The genes were cloned into the *Pichia* expression vector pPICZα A without the α-factor sequence and transformed into *P. pastoris* X33 cells. Transformants were checked for correct insertion of the *pmo* genes and replacement of the *aox1* gene in the *Pichia* genome by PCR with the standard primers 5´AOX and 3´AOX. A positive clone of each transformation was then chosen for enzyme production in a 7-L bioreactor. These cultivations were monitored for wet biomass, extracellular protein concentration, and PMO formation both by the PMO activity assay (Figure 4) and SDS-PAGE (Figure 5). After the batch and glycerol fed-batch phases for biomass build-up, the wet biomass concentration in the fermentation of PMO-01867, PMO-02916, PMO-03328 and PMO-08760 reached 349, 290, 371 and 342 g L^-1^, respectively. At this stage, no PMO activity could be detected and extracellular protein concentrations were very low (<0.1 g L^-1^). Cell growth slowed down considerably after induction with methanol, and expression of the target proteins could be observed both by an increase in extracellular protein concentration and PMO activity. All cultivations were well comparable with respect to biomass formation, and cell densities ranging from 388 to 447 g L^-1^ wet biomass were reached at the time of harvest. After around 120 hours of cultivation (PMO-01867, PMO-03328 and PMO-08760) or 145 hours (PMO-02916) of cultivation the extracellular protein concentrations reached 1.57, 1.82, 1.33 and 2.76 g L^-1^, respectively. PMO activities in the culture supernatants, however, showed that the expression levels of the target proteins varied considerably between PMO-01867 (0.46 ± 0.05 U L^-1^), PMO-03328 (0.72 ± 0.07 U L^-1^), PMO-08760 (1.46 ± 0.05 U L^-1^) and PMO-02916 (4.46 ± 0.04 U L^-1^). Accordingly, the fraction of PMO in total secreted protein was highest for PMO-02916 (28.5%), followed by PMO-08760 (25.7%) and PMO-01867 (19.9%) as calculated from the respective specific activities of these enzymes. Only 2.5% of the extracellular protein accounted for the small, non-glycosylated PMO-03328. These expression levels correlate well with the PMO bands seen on SDS-PAGE (Figure 5). The data for PMO activity and extracellular protein concentration at the time of harvest are summarized in Table 2. The fact that all four PMO enzymes were secreted and active confirms that *P. pastoris* is able to correctly process various *N. crassa* signal peptides. This strategy of using the native signal sequences is simpler than using the α-factor and subsequent proteolytic cleavage to guarantee the native N-terminal sequence of PMO [12]. The presence of the first N-terminal amino acid, a histidine, is essential since it participates in the coordination of the copper ion. An additional amino acid at the N-terminus or a loss of this histidine would render the protein inactive [4,15].

**Table 2:** Fermentation of PMO enzymes

**Enzyme**	**Harvest time**	**Wet biomass**	**Protein conc.**	**Vol. act.**	**Spec. act.**	**TTP conc.**^**a**^
	**[h]**	**[g L**^**-1**^**]**	**[g L**^**-1**^**]**	**[U L**^**-1**^**]**	**[U g**^**-1**^**]**	**[g L**^**-1**^**]**
PMO-01867	116.5	388	1.57	0.46	0.38	0.31
PMO-02916	145.0	447	2.76	4.46	1.59	0.79
PMO-03328	115.5	391	1.82	0.72	0.39	0.05
PMO-08760	119.5	441	1.33	1.46	1.05	0.34

^a^ Total Target Protein concentration was calculated from the total protein concentration using the specific activity of the purified PMO.

### Purification of recombinant PMOs

**Figure 6:**
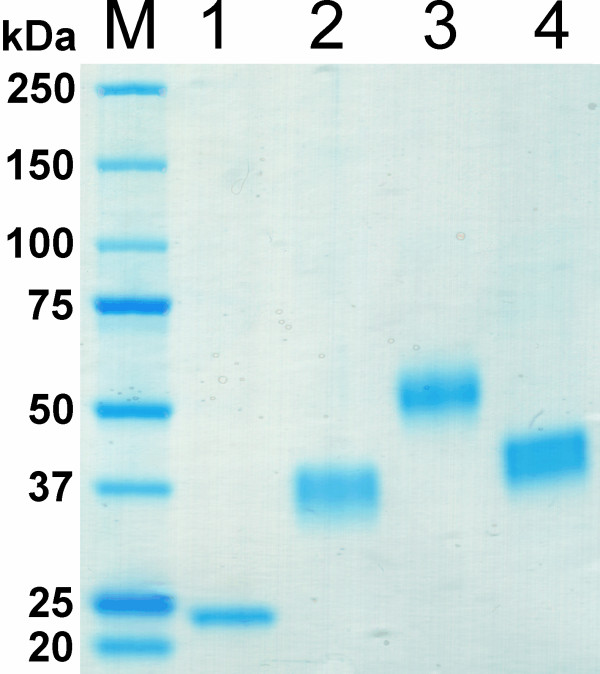
**SDS-PAGE of homogenously purified PMO enzymes.***M*, molecular marker (Bio-Rad), *Lane 1*, PMO-03328; *lane 2*, PMO-01867; *lane 3*, PMO-02916; *lane 4*, PMO-08760.

*N. crassa* PMOs were purified to homogeneity in a standardized three-step chromatographic purification procedure (Table 3) with the exception of PMO-02916, which already showed a single band on SDS-PAGE after two steps. The first step – hydrophobic interaction chromatography (HIC) – was used as a capturing step and increased the specific activity of the three glycosylated PMOs only around two-fold in combination with low yields especially of PMO-08760 and PMO-02916. The reason for this is incomplete binding of the PMOs even when applying high ammonium sulphate saturation and smearing during the elution. In contrast, the specific activity of non-glycosylated PMO-03328 was increased almost 7-fold with a yield of 76%. An obvious difference between the glycosylated and the non-glycosylated PMOs was the weaker binding of PMO-03328 to the HIC resin. While PMO-01867, PMO-02916 and PMO-08760 eluted at a conductivity of 53, 42 and 57 mS cm^-1^, respectively, PMO-03328 already eluted at 72 mS cm^-1^. The second step – anion exchange chromatography (AIEX) – took advantage of the very weak binding of all four PMOs to the selected resin. The conductivity of all PMO pools after HIC was reduced to <1.4 mS cm^-1^ and the pH was increased to 8.0 before application of the protein samples to the column. These conditions ensured the binding of a vast majority of yeast proteins while PMO was found in the flow-through. This product flow-through purification proved to be quick and efficient for all 4 PMOs. Subsequent gel filtration was used as polishing step and resulted in homogeneous protein preparations (Figure 6). Strict pooling of only the purest fractions resulted in the low overall yields. The purification procedure can certainly be improved by PMO specific modifications to this general, non-optimized purification procedure.

**Table 3:** Purification scheme of recombinant PMO enzymes

**Purification step**	**Total protein**^**a**^**[g]**	**Total activity [U]**	**Specific Activity [U g**^**-1**^**]**	**Purification [−fold]**	**Yield [%]**
**PMO-01867**					
Culture supernatant (2.5 L)	3.935	1.48	0.38	1	100
Phenyl Sepharose	0.536	0.39	0.74	2.0	27
Q-Source	0.211	0.25	1.18	3.2	17
Gel filtration	0.060	0.12	1.91	5.1	7.8
**PMO-02916**					
Culture supernatant (2.1 L)	5.800	9.21	1.59	1	100
Phenyl Sepharose	0.334	1.10	3.28	2.1	12
Q-Source	0.140	0.78	5.57	3.5	8
**PMO-03328**					
Culture supernatant (2.9 L)	5.275	2.08	0.39	1	100
Phenyl Sepharose	0.235	1.57	6.70	17.0	76
Q-Source	0.074	0.70	9.52	24.1	34
Gel filtration	0.007	0.11	15.76	39.9	5.4
**PMO-08760**					
Culture supernatant (2.3 L)	3.052	3.19	1.05	1	100
Phenyl Sepharose	0.158	0.30	1.87	1.8	9
Q-Source	0.047	0.12	2.54	2.4	4
Gel filtration	0.005	0.02	4.08	3.9	0.6

^a^All protein concentrations were measured with the Bradford assay.

The specific activities of the pure enzymes differ according to the method used to determine the protein concentration. When using the absorbance at 280 nm and the calculated molecular absorption coefficient, the specific activities are lower by a factor of 1.54 (PMO-01867), 2.1 (PMO-02916), 0.93 (PMO-03328) and 1.99 (PMO-08760) compared to the Bradford method for protein quantification with a BSA standard curve. This obviously reflects the inaccuracy of the Bradford assay for glycosylated proteins.

**Figure 7:**
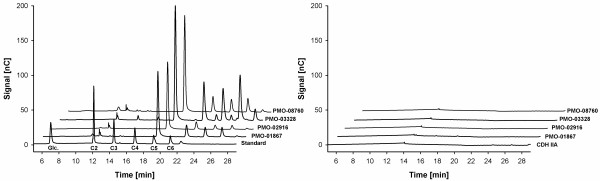
**HPLC chromatograms of PMOs incubated with cellulose and CDH and blank experiments without CDH.** Standards: Glc., glucose; C2, cellobiose; C3, cellotriose; C4, cellotetraose; C5, cellopentaose; C6, cellohexaose.

PMOs were incubated with cellulose and CDH/lactose as electron donor in an experiment to examine the lytic activity of the purified proteins on cellulose. HPLC analysis of the supernatant of the reaction mix showed the formation of cello-oligosaccharides in contrast to the blank reactions, where the reducing system CDH/lactose or the PMOs were omitted (Figure 7). The sample peaks could not fully be assigned to the peaks of the standard, because no standard for oxidized cello-oligosaccharides was available to us. However, the comparison with the negative controls and with results of similar experiments [3-5,12] not only shows that the purified proteins indeed are lytic PMOs, it also emphasized the importance of CDH as electron donor for the cellulolytic function.

### Molecular properties

Molecular masses were determined by SDS-PAGE (Figure 6). It is clearly visible that three of the four proteins are heavily glycosylated resulting in diffuse bands of considerably larger size than calculated from their amino acid sequences (Table 4). PMO-03328, the only PMO investigated in this study without a CBM is not glycosylated and shows a sharp band of the calculated size (23.7 ± 0.6 kDa). PMO-02916 is the largest protein with 57.5 ± 0.8 kDa and is 40.4% glycosylated, PMO-01867 and PMO-08760 (molecular masses of 39.6 ± 0.7 kDa and 41.4 ± 0.4 kDa) are glycosylated to a lesser extent, 17.5% and 22.2%, respectively. The size of PMO-08760 expressed in *P. pastoris* is very similar to the size of the protein from its native source *N. crassa*[3], suggesting a similar extent of glycosylation and no hyperglycosylation by the yeast. Deglycosylation with PNGase F under denaturing conditions had no visible effect on the appearance of the proteins on SDS-PAGE (data not shown), which has also been observed for other PMOs expressed in yeast [7,13]. PMO-02916 was the only investigated enzyme showing two N-glycosylation sequons (Table 4), and only prolonged deglycosylation with N-glycosidase F and α-mannosidase showed a decrease of its mass [18]; no N-glycosylation sites were predicted for PMO-01867 and PMO-08760. The three glycosylated PMOs show O-glycosylation sites, and since no loss of molecular mass was observed after PNGase treatment under denaturing conditions, we conclude that the glycosylation of the PMOs must be O-glycosylation, presumably at the Ser/Thr-rich linker connecting the enzyme to the C-terminal CBM [7]. The amino acid sequences of the PMOs with highlighted signal peptides, putative N- and O-glycosylation sites and CBMs are presented in Additional file 3.

**Table 4:** Molecular masses and glycosylation of PMO enzymes

**Enzyme**	**SDS-PAGE**	**Calculated**	**Glycan mass**	**Glycan mass**	**Putative glycosyl. sites**	
	**[kDa]**	**[kDa]**	**[kDa]**	**[%]**	***O*-Glyc.**	***N-*Glyc.**
PMO-01867	39.6 ± 0.7	32.7	6.9	17.5	5	0
PMO-02916	57.5 ± 0.8	34.3	23.2	40.4	27	2
PMO-03328	23.7 ± 0.6	23.2	0.0	0.0	5	0
PMO-08760	41.4 ± 0.4	32.2	9.2	22.2	16	0

**Figure 8:**
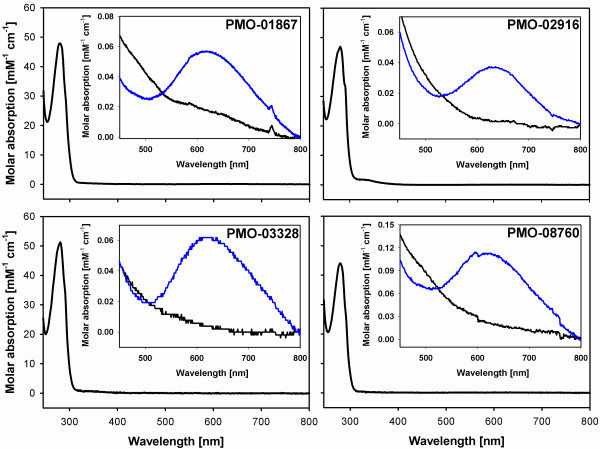
**Spectra of PMOs.** The weak absorption of the type-2 copper center is only visible in the inset. The small peaks around 615 – 650 nm disappear after reduction with ascorbate. Data for PMO-02916 was taken from Sygmund et al. [18].

The UV/VIS spectra of the purified PMOs (Figure 8) show peaks characteristic for a type-2 copper at around 620 nm. [19,20]. The absorbance is much weaker than that of a type-1 copper center, but clearly visible at high PMO concentration. The peaks vanished after reduction with ascorbate. The molar absorption coefficients and the Reinheitszahl (RZ) value for each PMO are presented in Table 5. The stability of the PMO enzymes was investigated by differential scanning calorimetry (DSC). The transition midpoint temperatures were roughly similar for all four proteins with 66.9, 63.0, 68.9 and 67.9°C for PMO-01867, PMO-02916, PMO-03328 and PMO-08760, respectively (Additional file 4). Thaw/freeze stability was also determined by three cycles of freezing in liquid nitrogen and thawing at room temperature. No loss of activity was observed.

**Table 5:** Spectral properties of investigated PMO enzymes

**Enzyme**	**ε**_**280**_	**λ**	**ε**_**type-2-Cu**_	**RZ value**
	**[mM**^**-1**^**cm**^**-1**^**]**	**[nm]**	**[mM**^**-1**^**cm**^**-1**^**]**	**[ε**_**280**_**/ε**_**type-2-Cu**_**]**
PMO-02916	46.91	630	0.037	1270
PMO-03328	51.13	620	0.062	830
PMO-01867	47.87	620	0.057	840
PMO-08760	43.86	615	0.113	390

The specific activities for the H_2_O_2_ side reaction of the PMOs were determined for CDH IIA as well as for ascorbate as reducing agent (Table 6). When using ascorbate these activities were remarkably similar for the three glycosylated PMOs with four to five U g^-1^ while PMO-03328 exhibited a three-fold higher activity. A similar picture was found for the assay with CDH IIA, however with more variation ranging from 1.07 ± 0.13 U g^-1^ (PMO-01867) to 15.46 ± 0.97 U g^-1^ for PMO-03328. These results could be a measure for varying affinity of the respective PMO for CDH IIA as electron donor. The much higher specific activity of PMO-03328 is partly owing to the lower enzyme mass and maybe to a higher H_2_O_2_ production rate, not necessarily reflecting higher PMO activity.

**Table 6:** Specific activities of PMO enzymes

**Enzyme**	**Reductant/Specific activity [U g**^**-1**^**]**	
	**CDH IIA**	**Ascorbate**
PMO-01867	1.07 ± 0.13	4.00 ± 0.18
PMO-02916	1.82 ± 0.04	4.47 ± 0.15
PMO-03328	15.46 ± 0.97	12.08 ± 0.67
PMO-08760	1.53 ± 0.19	4.84 ± 0.13

## Conclusions

*P. pastoris* was found to be a suitable expression host for glycosylated and non-glycosylated *N. crassa* PMO enzymes. A further advantage of this host is the correct processing of the *N. crassa* signal sequences of PMOs. This feature is especially important when considering that the active site of PMOs contains the N-terminal histidine. The PMO enzymes expressed in this study reduce oxygen and show typical type-2 copper absorption spectra, which supports the finding that these enzymes are in fact copper-containing PMOs. The newly developed CDH-driven activity assay allows fast PMO activity measurements in fermentation samples and is robust against interfering matrix components. With an easy assay for the target protein available, optimization of fermentation and purification conditions will be greatly simplified and should substantially improve the yields. Additionally, it allows the establishment of a high-throughput screening for improved activity enabling for example a screening for better producing *P. pastoris* clones. The second assay using ascorbate as reducing agent provides a tool to measure PMO activity independently from the interaction between PMO and its electron donor CDH, but it is too prone to interference to be used in crude enzyme samples such as fermentation media. Recombinant production of PMO enzymes should enable the formulation of more efficient, cost-effective enzyme solutions for biofuel production from lignocellulosic biomass.

## Methods

### Chemicals and microorganisms

All chemicals were of the highest purity grade available and were purchased from Sigma-Aldrich unless stated otherwise. Amplex Red (10-acetyl-3,7-dihydroxyphenoxazine) was purchased from VWR. Restriction endonucleases and T4 DNA ligase were obtained from Fermentas and were used as recommended by the manufacturer. Nucleic acid amplifications were done employing GoTAQ DNA Polymerase (Promega), dNTP mix, oligonucleotide primers (HVD Life Sciences, Vienna, Austria) and a C-1000 thermocycler (Bio-Rad Laboratories). *E. coli* strain DH5α (Invitrogen) was used for subcloning. The gene coding for a PMO enzyme from *N. crassa* (GenBank:NCU02916, *gh61-3*[21]) has been previously expressed and purified by Sygmund et al. [18]. To simplify the denomination and also prevent confusion caused by the newly discovered polysaccharide monooxygenase function, we suggest to name this enzyme PMO-02916 on the basis of its GenBank number, and the other PMOs accordingly PMO-01867 (GenBank:NCU01867, *gh61-10*[22], PMO-03328 (GenBank:NCU03328, *gh61-6*[21]), and PMO-08760 (GenBank:NCU08760, *gh61-5*[21]). The *N. crassa* genes encoding the PMO enzymes were codon-optimized for expression in *P. pastoris* (Additional file 5) and commercially synthesized by Invitrogen including their native signal sequences. *P. pastoris* strain X-33 and the vector pPICZα A are components of the Pichia Easy Select Expression System from Invitrogen. CDH IIA was produced and purified as previously reported [18].

### Amplex Red/horseradish peroxidase assay

The oxygen reactivity of PMOs was measured by a time resolved quantification of H_2_O_2_ formation in 96-well plates (total volume of 200 μL) using a Perkin Elmer EnSpire Multimode plate reader. All reactions were performed in 100 mM sodium phosphate buffer, pH 6.0 at 22°C. Based on preliminary studies ascorbate and CDH were used in concentrations of 30 μM and 0.3 μM (0.025 mg mL^-1^), respectively to prevent a limitation in the PMO reduction step. As electron donor for CDH 500 μM cellobiose was used. When ascorbate was used as reductant, it was added to a final concentration of 30 μM and enzyme assays were started by mixing 20 μL of the respective PMO with 180 μL of the ready-made assay solution containing 30 μM ascorbate, 50 μM Amplex Red and 7.14 U mL^-1^ peroxidase in 96-well plate wells. In reference experiments without PMO the background signal (H_2_O_2_ production by CDH) was measured and subtracted from the assays. When CDH was used as reductant, the PMO assays were started by mixing 20 μL of sample solution and 20 μL CDH solution with 160 μL of the reaction mix containing cellobiose, Amplex Red and peroxidase. Initial fluorescence scans of resorufin showed highest signal intensities and lowest interference with matrix compounds when using an excitation wavelength of 569 nm and an emission wavelength of 585 nm for the selected conditions. The stoichiometry of H_2_O_2_ conversion to resorufin formation is 1:1. By using a high concentration of Amplex Red (50 μM) the linearity of the detection reaction was ensured and the molar ratio of Amplex Red:H_2_O_2_ was always greater than 50:1 [22]. The H_2_O_2_ concentration in the assays was far below 1 μM, which leads to a linear concentration/activity response of horseradish peroxidase, which has a K_M_ for H_2_O_2_ of 1.55 μM. The high final activity of horseradish peroxidase (7.14 U mL^-1^) assures a fast conversion of the formed H_2_O_2_ and prevents the final reaction to be rate limiting. Additionally, it prevents the accumulation of H_2_O_2_, which could have detrimental effects on enzyme stability in the assay. The stability of resorufin fluorescence under these conditions was tested by addition of varying concentrations of hydrogen peroxide (0.1 – 5 μM) to the assay. A stable signal that remained constant throughout the measured period of 45 minutes was observed and maximum signal intensity was reached already during the mixing period before starting the assay. A linear relation between fluorescence and H_2_O_2_ concentrations in the range of 0.1 – 2 μM H_2_O_2_ was observed and the slope (28450 counts μmol^-1^) was used for the calculation of an enzyme factor to convert the fluorimeters readout (counts min^-1^), into enzyme activity. PMO activity was defined as one μmol H_2_O_2_ generated per minute under the defined assay conditions.

### Construction of *pmo* expression vectors for *P. pastoris*

The synthetic *pmo* genes were digested with the restriction enzymes *Bst*BI and *Xba*I and ligated into the equally treated vector pPICZα A using the Rapid DNA Ligation Kit from Fermentas. The procedures resulted in plasmids carrying genes encoding proteins with their native signal sequences cloned under the control of the methanol inducible AOX1 promoter. C-terminal tags for purification or antibody detection were omitted. The plasmids were linearized with the restriction enzyme *Sac*I and transformed into electro-competent *P. pastoris* cells. Transformants were grown on YPD plates (10 g L^-1^ yeast extract, 20 g L^-1^ peptone, 10 g L^-1^ glucose and 15 g L^-1^ agar) containing 100 mg L^-1^ zeocin.

### Enzyme production and purification

The enzymes were produced in a 7-L bioreactor (MBR) with a starting volume of 3 L Basal Salts Medium (21 mL L^-1^ H_3_PO_4_ (85%); 0.93 g L^-1^ CaSO_4_·2H_2_O; 14.9 g L^-1^ MgSO_4_·7H_2_O; 18.2 g L^-1^ K_2_SO_4_; 4.13 g L^-1^ KOH; 4% (v/v) glycerol) supplemented with 4.35 mL L^-1^ PTM_1_ trace salts (Invitrogen), 1 mL Antifoam 204 (Sigma) and 0.1 mM CuSO_4_. After sterilization, the pH of the medium was adjusted to pH 5.0 with 28% ammonium hydroxide and maintained at this level throughout the entire fermentation process. The fermentations were started by adding 400 mL preculture grown on YPD medium in 1-L baffled shaking flasks at 125 rpm and 30°C overnight. The cultivations were performed according to the Pichia Fermentation Process Guidelines of Invitrogen with some alterations. After depletion of glycerol in the batch medium the fed-batch phase was started with a constant feed of 36 mL h^-1^ 50% glycerol containing 12 mL L^-1^ PTM_1_ trace salts overnight to increase biomass. For induction the feed was switched to 100% methanol containing 12 mL L^-1^ PTM_1_ trace salts at an initial feed rate of 12 mL h^-1^ until the culture was fully adapted to methanol. Subsequently the feed rate was adjusted to keep the dissolved oxygen saturation constant at 4% at a constant air supply of 6 L min^-1^ and a stirrer speed of 800 rpm. After induction the cultivation temperature was reduced to 25°C. Samples were taken regularly and wet biomass, protein concentration and PMO activity were measured.

The fermentation broth was centrifuged at 6400 × g and 4°C for 30 min, and ammonium sulphate was slowly added to the clear culture supernatant to give a 30% saturated solution. Precipitate was removed by centrifugation (6400 × g; 20 min at 4°C) and the clear supernatant was loaded onto a 600-mL PHE-Sepharose Fast Flow column (chromatographic equipment and materials from GE Healthcare Biosciences) equilibrated with a 25 mM sodium acetate buffer, pH 5.0, containing 30% ammonium sulphate saturation. Proteins were eluted within a linear gradient from 30 to 0% ammonium sulphate within 3 column volumes and fractions were collected. Fractions containing the respective PMO were pooled and diafiltered with a hollow fiber cross-flow module (Microza UF module SLP-1053, 10 kDa cut-off, Pall Corporation). The diafiltered pools (conductivity < 1.4 mS cm^-1^) were applied to a 20-mL column packed with Q15-Source equilibrated with 20 mM TRIS/HCl buffer, pH 8.0. The flow-throughs were collected and contained the PMO enzymes. The solutions were concentrated, first by diafiltration with a Vivaflow cross-flow module (Millipore, cut-off 10 kDa) and subsequently by centrifugation in Amicon centrifugation tubes (Millipore, cut-off 10 kDa, 3200 x g, 15 min at 4°C). Size exclusion chromatography was done with a Superdex 75 column (Pharmacia Biotech) equilibrated with 20 mM TRIS/HCl, pH 8.0 buffer. Fractions containing pure PMO enzyme were pooled and stored at 4°C.

### Measurement of cellulolytic activity of PMOs

Microcrystalline cellulose was incubated with the PMOs and analyzed for soluble cello-oligosaccharides. The cellulose concentration in 50 mM sodium phosphate buffer, pH 6.0 was 25 mg mL^-1^ and PMOs were employed at a concentration of 5 mg g^-1^ cellulose. CDH IIA was used as reductant for PMO at a concentration of 2 mg g^-1^; cellulose and lactose (200 μM) were used as electron donors for CDH. A total volume of 400 μL was incubated at 30°C for 72 hours on a shaking incubator operated at 320 rpm and 90% humidity. The reaction tubes were sealed with an air permeable membrane to ensure continuous oxygen supply. Negative controls without CDH IIA and PMOs were performed under the same conditions. After incubation the samples were heated to 95°C for 10 min and protein precipitate and remaining cellulose was removed by centrifugation. Released cellooligosaccharides were detected by HPLC using a CarboPac PA100 Carbohydrate Column equipped with a guard column and an ED40 electrochemical detector (all equipment from DIONEX). The column was equilibrated with 150 mM NaOH (solution A) and reaction products were eluted by a linear gradient from 0 to 30% solution B (500 mM sodium carbonate in solution A) within 25 minutes at a flow rate of 0.5 mL min^-1^. The column was washed with 100% solution A for 5 minutes and re-equilibrated for 13 minutes with solution B before starting the next run.

### Protein concentrations

Protein contents of crude preparations or partially purified fractions were determined by the dye-binding method of Bradford using a pre-fabricated assay (Biorad Laboratories) and BSA as calibration standard. Protein concentrations of purified samples were measured based on their extinction coefficient at 280 nm (PMO) or 420 nm (CDH IIA).

### Spectral characterization

Spectra of homogenously purified PMO enzymes were recorded with a Hitachi U-3000 spectrophotometer at room temperature. Appropriately diluted enzymes (Abs ~1 at 280 nm) were measured from 250 to 800 nm at a scan speed of 60 nm min^-1^. To assess the extinction coefficients of the type-2 copper sites, spectra of concentrated enzymes were recorded from 450 to 800 nm at a scan speed of 30 nm min^-1^. Spectra of reduced species were obtained by addition of an excess of ascorbate to the cuvette. The molar absorption coefficients at 280 nm for all enzymes were calculated using the mature amino acid sequence and the program ProtParam ( http://web.expasy.org/protparam/).

### Deglycosylation

PMO enzymes (1.5 mg mL^-1^, 20 μL) were treated with PNGase F under denaturing conditions. The enzymes were mixed with 2 μL of glycoprotein denaturing buffer (80.5% sodium dodecylsulfate, 1% mercaptoethanol) and incubated at 99°C for 10 min. Then, 4 μL of 0.5 M sodium phosphate buffer, pH 7.5, 4 μL of nonylphenol (40%) and 1 μL of PNGase F were added to the reaction mix and incubated at 37°C for 1 hour. Glycosylation sequons were predicted using the servers NetNGlyc 1.0 ( http://www.cbs.dtu.dk/services/NetNGlyc/) and NetOGlyc 3.1 ( http://www.cbs.dtu.dk/services/NetOGlyc/).

### Gel electrophoresis

Mini-PROTEAN TGX precast gels (Bio-Rad Laboratories) with a gradient from 4 – 20% were used for SDS-PAGE analysis of purified enzymes and fermentation supernatants. Protein bands were visualized by staining with Bio-Safe Coomassie, and unstained Precision Plus Protein Standard was used for mass determination. All procedures were done according to the manufacturer’s recommendations (Bio-Rad Laboratories). Three independent gels were used for molecular mass determination of PMO enzymes.

### Differential scanning calorimetry

Thermodynamic stabilities of PMO enzymes were measured by differential scanning calorimetry (DSC) using a MicroCal VP-DSC instrument with an autosampler (MicroCal, Northampton, MA). Enzyme concentrations were adjusted to 1 mg mL^-1^ based on their molar extinction coefficients at 280 nm. All measurements were carried out in 100 mM phosphate buffer, pH 6.0. A linear temperature ramp from 20 to 100°C was applied at a scan rate of 1 K per min. Prior to all measurements, samples were degassed by continuous stirring *in vacuo* for 15 min. Obtained sample thermograms were corrected for the calorimeter baseline by subtracting a buffer blank that was scanned in the reference chamber. Transition midpoint temperatures (*T*_M_) of the enzymes were determined from the peak maximum of transition using the Origin 7.5 software (Origin Lab Corporation, Northampton, MA).

## Abbreviations

CDH: Cellobiose dehydrogenase; CMC: Carboxymethyl cellulose; GH61: Family 61 glycoside hydrolase; PASC: Phosphoric acid swollen cellulose; PMO: Polysaccharide monooxygenase.

## Competing interests

The authors declare that they have no competing interests.

## Authors' contributions

RK prepared the *P. pastoris* expression strains. RK and DB carried out the fermentation and purification of the PMOs. RL and DK developed the enzyme assay. DK performed the enzyme characterization and wrote part of the manuscript. RK coordinated the experiments and drafted the manuscript. DH and RL initiated, designed and coordinated the study and reviewed the manuscript. All authors have read and approved the final manuscript.

## Supplementary Material

Additional file 1 Stability of resorufin fluorescence at various concentrations. Click here for file

Additional file 2 **Background H**_**2**_**O**_**2**_** production by*Pichia*fermentation culture supernatants and permeates.** Click here for file

Additional file 3 **Amino acid sequences of PMO-01867, PMO-02916, PMO-03328 and PMO-08760.** Signal peptides are highlighted in green, putative N-glycosylation sites in yellow and putative O-glycosylation sites in red. CBMs are underlined. Click here for file

Additional file 4 DSC of PMO-01867, PMO-02916, PMO-03328 and PMO-08760. Click here for file

Additional file 5 **Codon optimized sequences of*pmo-01867*,*pmo-02916*,*pmo-03328*and*pmo-08760*.** Click here for file
