# Supplementary material for: Production of four Neurospora crassa lytic polysaccharide monooxygenases in Pichia pastoris monitored by a fluorimetric assay
Source: Biotechnol Biofuels. 2012 Oct 26;5:79. doi: 10.1186/1754-6834-5-79 (PMC3500269; doi:10.1186/1754-6834-5-79)
Supplement: Additional file 3 — Amino acid sequences of PMO-01867, PMO-02916, PMO-03328 and PMO-08760. Signal peptides are highlighted in green, putative N-glycosylation sites in yellow and putative O-glycosylation sites in red. CBMs are underlined. [file 1754-6834-5-79-S3.pdf]

```
                   10         20         30         40         50
           ....|....| ....|....| ....|....| ....|....| ....|....|
PMO-01867  MKSSLLVVLT AGLAVRDAIA HAIFQQLWVD GVDYGSTCNR LPTSNSPVTN

                   60         70         80         90        100
           ....|....| ....|....| ....|....| ....|....| ....|....|
PMO-01867  VGSRDVVCNA GTRGVSGKCP VKAGGTVTVE MHQQPGDRSC KSEAIGGAHW

                  110        120        130        140        150
           ....|....| ....|....| ....|....| ....|....| ....|....|
PMO-01867  GPVQIYLSKV SDASTADGSS GGWFKIFSDA WSKKSGGRVG DDDNWGTRDL

                  160        170        180        190        200
           ....|....| ....|....| ....|....| ....|....| ....|....|
PMO-01867  NACCGRMDVL IPKDLPSGDY LLRAEALALH TAGQSGGAQF YISCYQITVS

                  210        220        230        240        250
           ....|....| ....|....| ....|....| ....|....| ....|....|
PMO-01867  GGGSANYATV KFPGAYRASD PGIQINIHAV VSNYVAPGPA VVAGGVTKQA

                  260        270        280        290        300
           ....|....| ....|....| ....|....| ....|....| ....|....|
PMO-01867  GSGCIGCEST CKVGSSPSAV APGGKPASGG SDGNAPEVAE PSGGEGSPSA

                  310        320        330        340
           ....|....| ....|....| ....|....| ....|....| ....
PMO-01867  PGACEVAAYG QCGGDQYSGC TQCASGYTCK AVSPPYYSQC APTS


                   10         20         30         40         50
           ....|....| ....|....| ....|....| ....|....| ....|....|
PMO-02916  MKTGSILAAL VASASAHTIF QKVSVNGADQ GQLKGIRAPA NNNPVTDVMS

                   60         70         80         90        100
           ....|....| ....|....| ....|....| ....|....| ....|....|
PMO-02916  SDIICNAVTM KDSNVLTVPA GAKVGHFWGH EIGGAAGPND ADNPIAASHK

                  110        120        130        140        150
           ....|....| ....|....| ....|....| ....|....| ....|....|
PMO-02916  GPIMVYLAKV DNAATTGTSG LKWFKVAEAG LSNGKWAVDD LIANNGWSYF

                  160        170        180        190        200
           ....|....| ....|....| ....|....| ....|....| ....|....|
PMO-02916  DMPTCIAPGQ YLMRAELIAL HNAGSQAGAQ FYIGCAQINV TGGGSASPSN

                  210        220        230        240        250
           ....|....| ....|....| ....|....| ....|....| ....|....|
PMO-02916  TVSFPGAYSA SDPGILINIY GGSGKTDNGG KPYQIPGPAL FTCPAGGSGG

                  260        270        280        290        300
           ....|....| ....|....| ....|....| ....|....| ....|....|
PMO-02916  SSPAPATTAS TPKPTSASAP KPVSTTASTP KPTNGSGSGT GAAHSTKCGG

                  310        320        330        340        350
           ....|....| ....|....| ....|....| ....|....| ....|....|
PMO-02916  SKPAATTKAS NPQPTNGGNS AVRAAALYGQ CGGKGWTGPT SCASGTCKFS

           ....|....
PMO-02916  NDWYSQCLP
```

```
                   10         20         30         40         50
           ....|....| ....|....| ....|....| ....|....| ....|....|
PMO-03328  MLPSISLLLA AALGTSAHYT FPKVWANSGT TADWQYVRRA DNWQNNGFVD

                   60         70         80         90        100
           ....|....| ....|....| ....|....| ....|....| ....|....|
PMO-03328  NVNSQQIRCF QSTHSPAQST LSVAAGTTIT YGAAPSVYHP GPMQFYLARV

                  110        120        130        140        150
           ....|....| ....|....| ....|....| ....|....| ....|....|
PMO-03328  PDGQDINSWT GEGAVWFKIY HEQPTFGSQL TWSSNGKSSF PVKIPSCIKS

                  160        170        180        190        200
           ....|....| ....|....| ....|....| ....|....| ....|....|
PMO-03328  GSYLLRAEHI GLHVAQSSGA AQFYISCAQL SITGGGSTEP GANYKVSFPG

                  210        220        230
           ....|....| ....|....| ....|....| .
PMO-03328  AYKASDPGIL ININYPVPTS YKNPGPSVFT C
```

```
                   10         20         30         40         50
           ....|....| ....|....| ....|....| ....|....| ....|....|
PMO-08760  MRSTLVTGLI AGLLSQQAAA HATFQALWVD GADYGSQCAR VPPSNSPVTD

                   60         70         80         90        100
           ....|....| ....|....| ....|....| ....|....| ....|....|
PMO-08760  VTSNAMRCNT GTSPVAKKCP VKAGSTVTVE MHQSHPPVPT LTYKQQANDR

                  110        120        130        140        150
           ....|....| ....|....| ....|....| ....|....| ....|....|
PMO-08760  SCSSEAIGGA HYGPVLVYMS KVSDAASADG SSGWFKIFED TWAKKPSSSS

                  160        170        180        190        200
           ....|....| ....|....| ....|....| ....|....| ....|....|
PMO-08760  GDDDFWGVKD LNSCCGKMQV KIPSDIPAGD YLLRAEVIAL HTAASAGGAQ

                  210        220        230        240        250
           ....|....| ....|....| ....|....| ....|....| ....|....|
PMO-08760  LYMTCYQISV TGGGSATPAT VSFPGAYKSS DPGILVDIHS AMSTYVAPGP

                  260        270        280        290        300
           ....|....| ....|....| ....|....| ....|....| ....|....|
PMO-08760  AVYSGGSSKK AGSGCVGCES TCKVGSGPTG TASAVPVAST SAAAGGGGGG

                  310        320        330        340
           ....|....| ....|....| ....|....| ....|....| ..
PMO-08760  GSGGCSVAKY QQCGGTGYTG CTSCASGSTC SAVSPPYYSQ CV
```
