# Supplementary figures and images for: Production of four Neurospora crassa lytic polysaccharide monooxygenases in Pichia pastoris monitored by a fluorimetric assay
Source: Biotechnol Biofuels. 2012 Oct 26;5:79. doi: 10.1186/1754-6834-5-79 (PMC3500269; doi:10.1186/1754-6834-5-79)

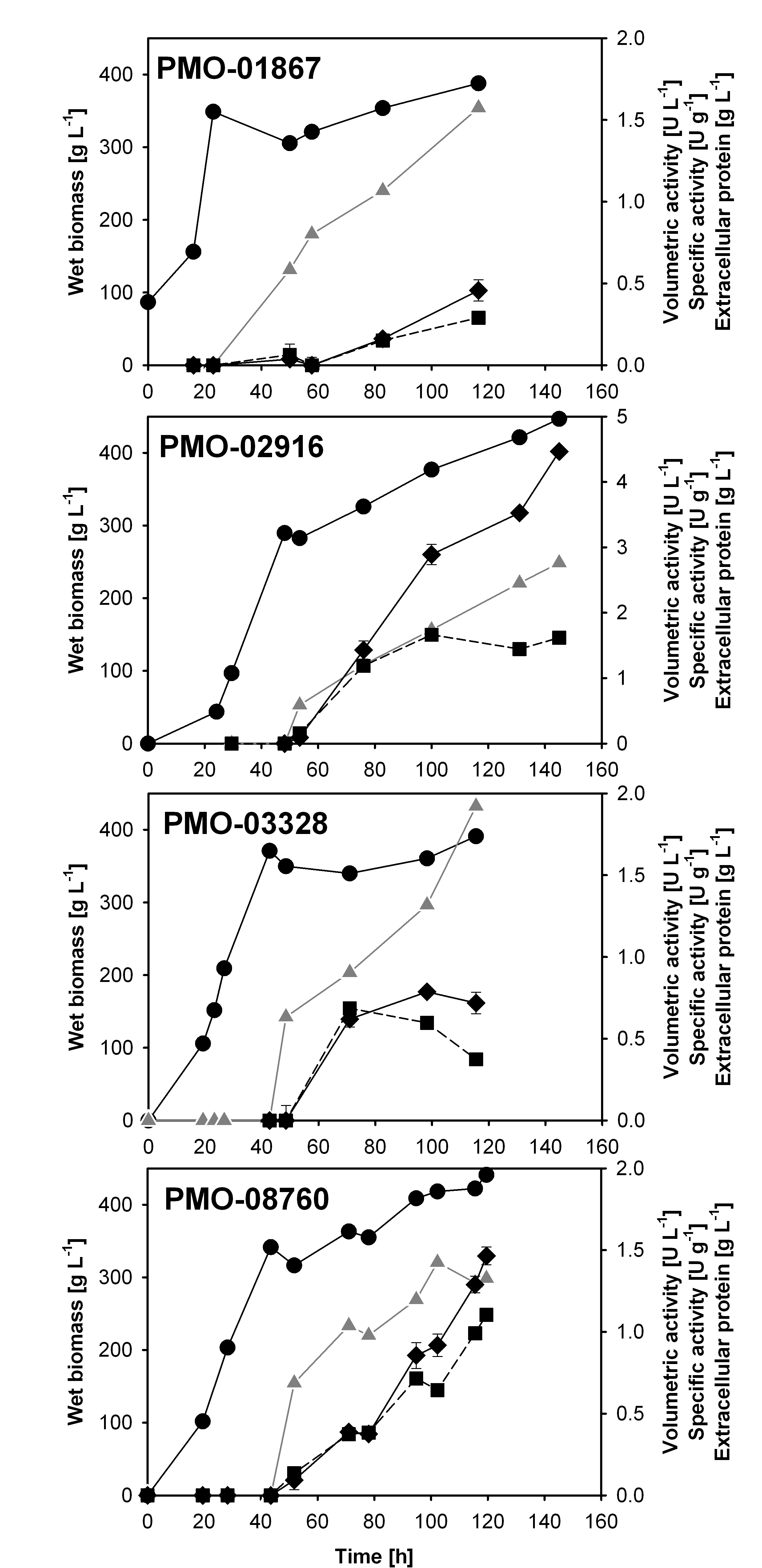

Supplement: Additional file 4 — DSC of PMO-01867, PMO-02916, PMO-03328 and PMO-08760. [file 1754-6834-5-79-S4.tiff]
